# Mechanisms Associated to Nitroxyl (HNO)-Induced Relaxation in the Intestinal Smooth Muscle

**DOI:** 10.3389/fphys.2020.00438

**Published:** 2020-06-03

**Authors:** Mirko Gastreich-Seelig, Marcel Jimenez, Ervice Pouokam

**Affiliations:** ^1^Institute for Veterinary Physiology and Biochemistry, Justus-Liebig-University Giessen, Giessen, Germany; ^2^Department of Cell Biology, Physiology and Immunology and Neurosciences Institute, Universitat Autònoma de Barcelona, Barcelona, Spain

**Keywords:** Ca^2+^, gasotransmitter, membrane potential, motility, nitroxyl (HNO), small conductance Ca^2+^-activated K^+^ channels (SKca), soluble guanylate cyclase

## Abstract

The pharmacological properties of nitroxyl (HNO) donors in the gastrointestinal tract are unknown. We investigated the properties of this molecule in the regulation of gastrointestinal contractility focusing on its possible interaction with other gaseous signaling molecules such as NO and H_2_S. Organ bath, Ca^2+^ imaging, and microelectrode recordings were performed on rat intestinal samples, using Angeli’s salt as HNO donor. Angeli’s salt caused a concentration-dependent relaxation of longitudinal or circular muscle strips of the ileum and the proximal colon. This relaxation was strongly inhibited by the Rho-kinase inhibitor Y-27632 (10 μM), by the reducing agent DTT or by the inhibitor of soluble guanylate cyclase (sGC) ODQ (10 μM) alone or in combination with the inhibitors of the endogenous synthesis of H_2_S β-cyano-L-alanine (5 mM) and amino-oxyacetate (5 mM). Preventing endogenous synthesis of NO by the NO synthase inhibitor L-NAME (200 μM) did not affect the relaxation induced by HNO. HNO induced an increase in cytosolic Ca^2+^ concentration in colonic myocytes. It also elicited myocyte membrane hyperpolarization that amounted to −10.6 ± 1.1 mV. ODQ (10 μM) and Apamin (1 μM), a selective inhibitor of small conductance Ca^2+^-activated K^+^ channels (SKca), strongly antagonized this effect. We conclude that HNO relaxes the gastrointestinal tract musculature by hyperpolarizing myocytes via activation of the sGC/cGMP pathway similarly to NO, not only inhibiting the RhoK and activating MLCP as do both NO and H_2_S but also increasing cytosolic Ca^2+^ for activation of SK_*C*__*a*_ contributing to hyperpolarization.

## Introduction

Gastrointestinal motility is regulated by enteric neurotransmitters inducing contraction and relaxation of the smooth muscle cells. Excitatory neurotransmitters such as acetylcholine (ACh) elicit smooth muscle cell depolarization and contraction, whereas inhibitory neurotransmitters cause smooth muscle hyperpolarization and relaxation. Inhibitory junction potentials are mainly due to ATP (or a related purine) and NO, causing a transient fast inhibitory junction potential (IJPf) followed by a sustained IJP (IJPs), respectively. Hydrogen sulfide (H_2_S) and nitric oxide (NO) are two signaling molecules in the enteric nervous system with inhibitory effects on gastrointestinal smooth muscle (Schemann, 2005; Wood, 2008; Gallego et al., 2014; Jimenez et al., 2014).

These two signaling molecules exert their actions on smooth muscles via different mechanisms. In canine colonic smooth muscle cells, exogenous NO caused accumulation of cytosolic cGMP (Shuttleworth et al., 1993). Endogenous NO either depresses the release of ACh from interneurons in descending enteric pathways or facilitates ACh release in the ascending pathways; both mechanisms involve soluble guanylate cyclase (sGC) (Smith and McCarron, 1998). The relative contribution of these pathways in the action of NO may differ depending on either the species or the segment of the gastrointestinal tract concerned. In contrast, H_2_S does activate the opening of ATP-sensitive K^+^ (K_*ATP*_) channels and the closure of voltage-dependent K^+^ channels (K_*v*_) in guinea pig gastric antrum myocytes (Zhao et al., 2009). In human and rat and mouse colon and jejunum, relaxant effects of H_2_S are dependent on apamin-sensitive small conductance Ca^2+^-activated K (SK) and on K_*ATP*_ channels (Gallego et al., 2008). In addition, activation of the myosin light chain phosphatase (MLCP) may account for the relaxing properties of H_2_S as shown in murine gastric fundus (Dhaese and Lefebvre, 2009).

A third gaseous signaling molecule, nitroxyl (HNO), is getting more attention (Irvine et al., 2008; Pouokam et al., 2013; Eberhardt et al., 2014; Cowart et al., 2015). The most used HNO donor is Angeli’s salt, which decomposes spontaneously at 37°C in physiological solutions at neutral pH to produce equimolar amounts of nitrite and HNO with a half-life of about 2.3 min. The formed HNO may in turn dimerize with elimination of water producing inert N_2_O (Katrina et al., 2005).

Its positive inotropic and lusitropic effects are important therapeutic actions in the heart. So far, HNO properties have been investigated mainly in the cardiovascular system (Irvine et al., 2008; Eberhardt et al., 2014). In the gastrointestinal tract, upon repetitive application in Ussing chambers, the HNO-donor Angeli’s salt induced a Ca^2+^-dependent Cl^–^ secretion without desensitization (Pouokam et al., 2013). The mechanism of action clearly differed from that of the secretion induced by its sibling NO because it did not depend on the activity of the sGC, which is the prototypical site of action of NO. In contrast, HNO-evoked secretion was blocked by indomethacin, suggesting that cyclooxygenase metabolites such as prostaglandins mediate the response, to which an activation of the basolateral Na^+^-K^+^-ATPase, Ca^2+^-dependent K^+^, and ATP-sensitive K^+^ channels contributed (Pouokam et al., 2013).

Recent studies showed a cross-talk of H_2_S and NO signaling pathways (Yong et al., 2011; Filipovic et al., 2013; Miljkovic et al., 2013; Eberhardt et al., 2014; Zhou et al., 2017; An et al., 2019). It is known that sodium nitroprusside (SNP) does release NO in ambient light but not in the dark and that combining equimolar SNP and H_2_S does not allow NO release in ambient light (Filipovic et al., 2013). It has been shown that a direct interplay between SNP and H_2_S leads to HNO production (Filipovic et al., 2013). Both the NO donor SNP and H_2_S [delivered by sodium sulfide (Na_2_S)] undergo a fast chemical reaction at pH 7.4 and under aerobic conditions, forming the intermediate [(CN)_5_FeN(O)SH]^3–^. In a second reaction step, the coordinated HSNO/SNO^–^ is reduced by H_2_S, which becomes oxidized to disulfide. The resulting disulfides may undergo further oxidations to polysulfides. The product of this second step is the intermediate [(CN)_5_Fe(HNO)]^3–^, which is converted to thiocyanate products after reaction with polysulfides and elimination of nitroxyl (HNO) (Filipovic et al., 2013; Ivanovic-Burmazovic and Filipovic, 2019).

Such a conversion of cyanide into thiocyanate being catalyzed by rhodaneses *in vivo* allowing therefore for the elimination of toxic cyanide (for review, see Mueller, 2006; Cipollone et al., 2007). Thus, the combination NO/H_2_S and therefore HNO appears therapeutically very interesting, as it is less toxic than the combination SNP/thiosulfate used for example in acute hypertensive crises to regulate blood pressure (Butler Anthony and Feelisch, 2008; Filipovic et al., 2013). An interaction between NO and H_2_S in the presence of oxygen may lead to nitrous oxide (N_2_O), sulfur, and water as described previously (Kurtenacker and Löschner, 1938; Koppenol and Bounds, 2017) and shown in the following Reaction (1). The single-step formation of N_2_O being unlikely, the latter may result from dimerization of HNO as shown by Reaction (2) (Kohout and Lampe, 1965; Bartberger et al., 2002).

(1) $${\text{H}}_{2}\,\text{S}+2\,\text{NO}\overset{\left[{\text{O}}_{2}\right]}{⟶}{\text{N}}_{2}\,\text{O}+\text{S}+{\text{H}}_{2}\,\text{O}$$

(2) $$2\,H\,N\,O⟶{\text{N}}_{2}\,\text{O}+{\text{H}}_{2}\,\text{O}$$

It has been recently confirmed that NO and H_2_S cooperatively generate HNO in cells, as for example, a strong increase in the HNO-sensor CuBOT1 was observed in dorsal root ganglia (DRG) neurons only when both gases were simultaneously applied (Eberhardt et al., 2014). Similarly, this interplay was revealed with new generations of HNO-sensitive dyes such as TP-Rho-HNO (Zhou et al., 2017) or HNOCL-1 (An et al., 2019) as endogenous NO may be transformed to HNO by H_2_S (Eberhardt et al., 2014), some effects of H_2_S may be ascribed to HNO.

Since HNO presents interesting clinical properties, we aimed to investigate the properties of this agent in the regulation of intestinal contractility, revealing its specific characters with regard to the well-known signaling molecules NO and H_2_S. To our knowledge, this is the first report studying neuromuscular actions of HNO in the GI tract.

## Materials and Methods

### Animals

Female and male Wistar (160–220 g) or Sprague–Dawley rats (12–18 weeks old) were used. The animals were bred and housed at the Institute for Veterinary Physiology and Biochemistry of the Justus-Liebig-University Giessen (Wistar rats) or at the Institute of Veterinary Physiology of the Universitat Autònoma de Barcelona (Sprague–Dawley rats) at an ambient temperature of 22.5°C and air humidity of 50–55% on a 12 h/12 h light–dark cycle with free access to water and food until the time of the experiment. The animals were anesthetized with CO_2_ and killed by exsanguination. The Sprague–Dawley rats were used for microelectrode experiments and the Wistar rats for others. Experiments were approved by the named animal welfare officer of the Justus Liebig University (administrative number 577_M) or by the Ethics Committee of the Universitat Autònoma de Barcelona (administrative number MJF-eut/01) and performed according to the German and European animal welfare law.

### Isometric Force Measurements

For isometric force measurements, the muscle strips were obtained as follows: the abdomen of euthanized animals was opened, the ileum and colon were collected, and the lumen was carefully cleaned. After a short period in ice-cold buffer gassed with 5% (*v*/*v*) CO_2_/95% (*v*/*v*) O_2_, the tissues were cut into 1.5 cm long pieces, which were then fixed in the organ bath. The chamber was filled with warm (37°C) and gassed (5% CO_2_/95% O_2_, *v*/*v*) buffer solution. For longitudinal muscle strips, the pretension was set at 1.5 g, and after an equilibrium period of at least 15 min, the tension was lowered to 1 g. For circular muscle strips (1 × 0.5 cm), the tension was continuously set at 1 g. The baseline was measured for 5 min before administration of any drug. As viability control, 10 μM carbachol and/or 30 mM KCl were administered at the end of each experiment.

Isometric force was measured via a BioAmp-04/8 amplifier system and sampled via an A/D-converter with a sampling rate of 1 Hz (Föhr Medical Instruments, Seeheim, Germany). For data analysis, the baseline just prior administration of a drug was measured as mean over 1 min. To calculate the maximal relaxation induced by Angeli’s salt, the maximal reduction in muscle tone within 5 min after administration of the agonist was calculated and expressed as difference to the baseline just prior administration of Angeli’s salt (Δ Force; see, e.g., Figure 4). As the action of Angeli’s salt on muscle tone was only transient, in addition the changes in the area under the curve (AUC) over a 3-min interval (g⋅180 s) before and after administration of the HNO donor was calculated (see inset in Figure 3). To determine the frequency of phasic spontaneous contractions, first time derivatives (d*g*/d*t*) were calculated. Only waves passing a threshold set at 0.1 g s^–1^ were counted as contraction during a 1-min period before (control) and 3 min after administration of HNO. The calculated frequency was expressed as contractions/min (cpm). To prove whether Angeli’s salt effects were due to HNO and not nitrite, a control with decomposed Angeli’s salt was performed (see Supplementary Figure S1) as previously done (Paolocci et al., 2001; Fukuto et al., 2008) to prove that the decomposed form has different biological properties. The experiments were conducted in ambient light. For nitric oxide (NO) delivery, SNP was used, as it is known to release NO in ambient light (Filipovic et al., 2013).

### Isolation and Identification of Myocytes

Ca^2+^ imaging experiments were performed at isolated myocytes from the proximal colon. The longitudinal muscle layer (devoid of mesenterium) from the colon was removed and cut in small pieces of about 0.1 × 0.1 cm. These pieces were collected and transferred into the digestion solution consisting of collagenase type II (0.5 mg ml^–1^) and trypsin inhibitor (0.25 mg ml^–1^) in Hank’s balanced salt solution (Ca^2+^- and Mg^2+^-free). If cells from the circular muscle layer had to be prepared, after stripping away the longitudinal muscle layer, the mucosa layer was scrapped off using the edge of a glass slide; the remaining tissue was minced, then proceeded as with the longitudinal layer. The tissues were then enzymatically digested for 30 min at 37°C then vortexed for 10 s and centrifuged 2,000 rpm for 3 min. The supernatant was discarded, and the digestion solution was added to the pellet. After shortly mixing, a second incubation period at 37°C for 30 min followed. The preparation was then vortexed for 5 s and centrifuged at 1,500 rpm for 3 min. The supernatant was discarded, and the myocytes were resuspended in Dulbecco’s modified Eagle’s medium (DMEM) F12 medium supplemented with fetal calf serum until the beginning of measurements. At the end of Ca^2+^ imaging experiments, tested myocytes were additionally morphologically identified. After fixation for 10 min in 4% (*w*/*v*) paraformaldehyde (PFA) at 37°C, their actin filaments were stained by fluorescein isothiocyanate (FITC)-conjugated phalloidin (800 nM) first at room temperature in the presence of 0.1% (*v*/*v*) Triton-X100, then overnight at 4°C. Nuclei were counterstained with 4′,6-diamidino-2-phenylindole (DAPI) for 5 min. Staining was repeated at least three times with cells from different animals. Myocyte staining was analyzed using a fluorescence microscope (80i, Nikon, Düsseldorf, Germany). Digital images were taken with black and white camera (DS-2M B/Wc) using NS Elements 2.30 software.

### Microelectrode Recordings

For microelectrode recordings, the colon of Sprague–Dawley rats was removed and placed in carbogenated (95% O_2_ and 5% CO_2_, *v*/*v*) Krebs solution, then opened along the mesenteric border. The mucosal and submucosal layers were gently removed and 3 × 5 mm muscle strips were cut in a circular direction.

Electrophysiological experiments were performed with colonic strips pinned in a Sylgard-coated chamber with the circular muscle layer facing upwards. The tissue was continuously perfused with carbogenated Krebs solution at 37 ± 1°C and allowed to equilibrate for 1 h. Phentolamine, propranolol, and atropine (all at 1 μM) were added to block α- and β-adrenoceptors and muscarinic receptors, respectively, so that the action of Angeli’s salt could be measured under non-adrenergic non-cholinergic (NANC) conditions. To obtain stable microelectrode impalements, nifedipine 1 μM was added to abolish mechanical activity. Circular smooth muscle cells were impaled using glass microelectrodes filled with 3 M KCl (30–60 MΩ of tip resistance). Membrane potential was measured using a standard Duo 773 electrometer (WPI Inc., Sarasota, FL, United States). Tracings were displayed on an oscilloscope (Racal-Dana Ltd., Windsor, United Kingdom) and simultaneously digitalized (100 Hz) with a PowerLab 4/30 system and Chart 5 software for Windows (both from ADInstrument, Castle Hill, NSW, Australia). IJPs were elicited by electrical field stimulation (EFS) using two silver chloride plates placed 1.5 cm apart perpendicular to the longitudinal axis of the preparation. The protocol consisted of single pulse trains of EFS (0.4 ms pulse duration) at supramaximal voltage (30–40 V). The resting membrane potential (RMP) and both the amplitude as well as the duration of the IJP were measured and compared before and after drug incubation.

### Ca^2+^ Imaging

The myocytes suspension was spun down at 1,500 rpm for 1 min. After discarding the supernatant, the cells were resuspended in warm (37°C) Tyrode solution containing the Ca^2+^-sensitive fluorescent dye fura-2 AM (6 μM; Life Technologies, Darmstadt, Germany) and pluronic acid (1.2 mg L^–1^; Life Technologies, Darmstadt, Germany) and incubated for 1 h at 37°C. After incubation, 30 μl of the myocyte suspension was spread onto a poly-L-lysine-coated coverslip and incubated at room temperature for 15 min, before it was washed carefully. The coverslip was then mounted in the experimental chamber with a volume of about 3 ml. The preparation was superfused hydrostatically with warm Tyrode solution (37°C). Perfusion was stopped only if the drug applied had to be applied via a pipette. The perfusion rate was about 2 ml min^–1^. Changes in the cytosolic Ca^2+^ concentration were monitored as changes in the fura-2 ratio (R; emission at an excitation wave length of 340 nm divided by the emission at an excitation wave length of 380 nm). Experiments were carried out on an inverted microscope (Olympus IX-50; Olympus, Hamburg, Germany), equipped with an epifluorescence setup and an image analysis system (Till Photonics, Martinsried, Germany). Several regions of interest, each with the size of about one cell, were selected. The emission above 420 nm was measured from the regions of interest. Data were sampled at 0.2 Hz. The baseline in the fluorescence ratio of fura-2 was measured for several minutes before drugs were administered.

### Solutions and Reagents

The organ bath Parsons solution consisted of (in mM): NaCl 107, KCl 4.5, NaHCO_3_ 25, Na_2_HPO_4_ 1.8, NaH_2_PO_4_ 0.2, CaCl_2_ 1.25, MgSO_4_ 1, and glucose 12. The solution was gassed with carbogen (5% CO_2_ in 95% O_2_, *v*/*v*); pH was 7.4. The composition of the Krebs solution was (in mM): glucose 10.1, NaCl 115.5, NaHCO_3_ 21.9, KCl 4.6, NaH_2_PO_4_ 1.1, CaCl_2_ 2.5, and MgSO_4_ 1.2 bubbled with a carbogen (pH 7.4). The myocytes were dissociated in Ca^2+^- and Mg^2+^-free Hank’s balanced salt solution (HBSS, Life Technologies, Paisley, United Kingdom). For superfusion of the isolated myocytes during the imaging experiments, a Tyrode solution was used (in mM): NaCl 140, KCl 5.4, CaCl_2_ 1.25, MgSO_4_ 1, HEPES 10, and glucose 12.2; pH was 7.4. For additional identification of myocytes, FITC-conjugated phalloidin (Sigma-Aldrich, St Louis, United States) and Roti-Mount Fluor DAPI (Carl Roth, Karlsruhe, Germany) were used.

Angeli’s salt (Cayman Chemical, Ann Arbor, MI, United States) was dissolved in 0.01 N NaOH. Indomethacin and Nifedipine were dissolved in 96% (*v*/*v*) ethanol. Calyculin A (New England, Ipswitch, United States), glibenclamide, and ODQ (Tocris, Bristol, United Kingdom) were dissolved in dimethyl sulfoxide (DMSO). Tetrodotoxin (TTX) (Alomone Labs, Jerusalem, Israel) was dissolved in citrate buffer. Amino-oxyacetate (AOAA), apamine (Alomone Labs, Jerusalem, Israel), atropine sulfate, carbachol, β-cyano-l-alanine (CLA), MRS2500 (Tocris, Bristol, United Kingdom), 1,4-dithiothreitol (DTT), N_ω_-nitro-L-arginine methylester hydrochloride (L-NAME), phentolamine, propranolol, sodium hydrogen sulfide (NaHS), sodium nitroprusside (SNP; Enzo Life, Lausen, Switzerland), and Y-27632 (Tocris, Bristol, United Kingdom) were dissolved in aqueous solutions. If not stated explicitly, drugs were purchased from Sigma Aldrich, St Louis, United States.

### Data Analysis and Statistics

In general, results are given as means ± standard error of the mean (SEM). The number of investigated tissues or cells is indicated “*n*.” For imaging experiments (Ca^2+^ imaging and immunostaining), the same protocol had to be run at least three times on different days and with different animals. When means of several groups had to be compared, an analysis of variance was performed followed by Bonferroni or Tuckey *post-hoc* test. For the comparison of two groups, either a paired or an unpaired Student’s *t*-test or a Mann–Whitney *U*-test was applied. Statistical analysis was performed with GraphPad Prism 6. Data were considered significant when *P* < 0.05.

## Results

### Nitroxyl Relaxes Gastrointestinal Muscle

**FIGURE 1:**
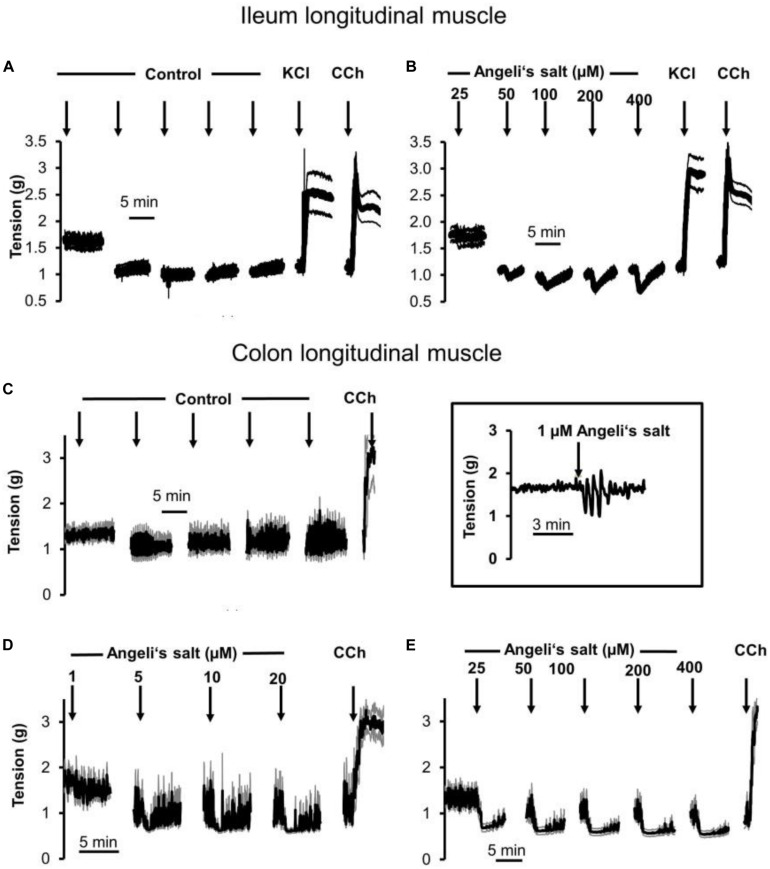
Relaxation induced by different concentrations of Angeli’s salt (1–400 μM, arrows) on longitudinal segments of **(B)** rat ileum or **(D,E)** proximal colon and **(A)** or **(C)** time-dependent control experiments, where only the solvent of Angeli’s salt was administered. The inset (middle) shows an original tracing of an individual colonic longitudinal muscle strip with a transient relaxation induced by 1 μM Angeli’s salt, which is hard to recognize in the ensemble average depicted in **(D)** resulting from the averaging of eight muscle strips responding asynchronously with a short relaxation induced by the HNO donor. Line interruptions are caused by omitting washing periods of about 10 min, where the content of the organ bath was exchanged three times before the next concentration of Angeli’s salt was administered. KCl (30 mM) and or carbachol (CCh; 10 μM) were used to check tissue viability. Values are means of individual tracings (black lines) ± SEM (gray lines), *n* = 5–11. For statistics **(B,D,E)** are plotted in Figures 3A–D, respectively.

The effects of Angeli’s salt were due to HNO, as the decomposed form of the salt was inefficient (see Supplementary Figure S1). The HNO donor Angeli’s salt induced a reduction in the basal tone, a decrease in spontaneous contractions leading to a reduction in the AUC of all three intestinal muscle preparations tested. In ileal longitudinal muscle, concentrations above 25 μM induced a transient fall in muscle tone, which lasted for 3–5 min (Figure 1B). Concentrations lower than 25 μM were ineffective (data not shown). Muscle tone remained stable in time-dependent controls treated with the solvent only (Figure 1A). In addition, contractions induced by KCl (30 mM) or carbachol (10 μM) were unaffected after washout of the HNO donor (Figures 1A,B). Concentration–response curves based either on the amplitude of the maximal relaxation induced by the HNO donor (Figure 3A) or on the integrated change in muscle tone over a 3-min period (Figure 3B) revealed a flat concentration dependence of the effect of Angeli’s salt.

In addition, longitudinal muscle from the proximal colon responded with a concentration-dependent relaxation (Figures 1D,E, 3C,D), which was not observed in parallelly performed time-dependent control experiments (Figure 1C). In contrast to the ileum, the longitudinal muscle strips from the colon responded to the HNO donor at much lower concentrations. However, with higher concentrations of Angeli’s salt, the duration of the induced relaxation was prolonged. Contraction induced by carbachol (10 μM) was unaffected (Figures 1D,E).

**FIGURE 2:**
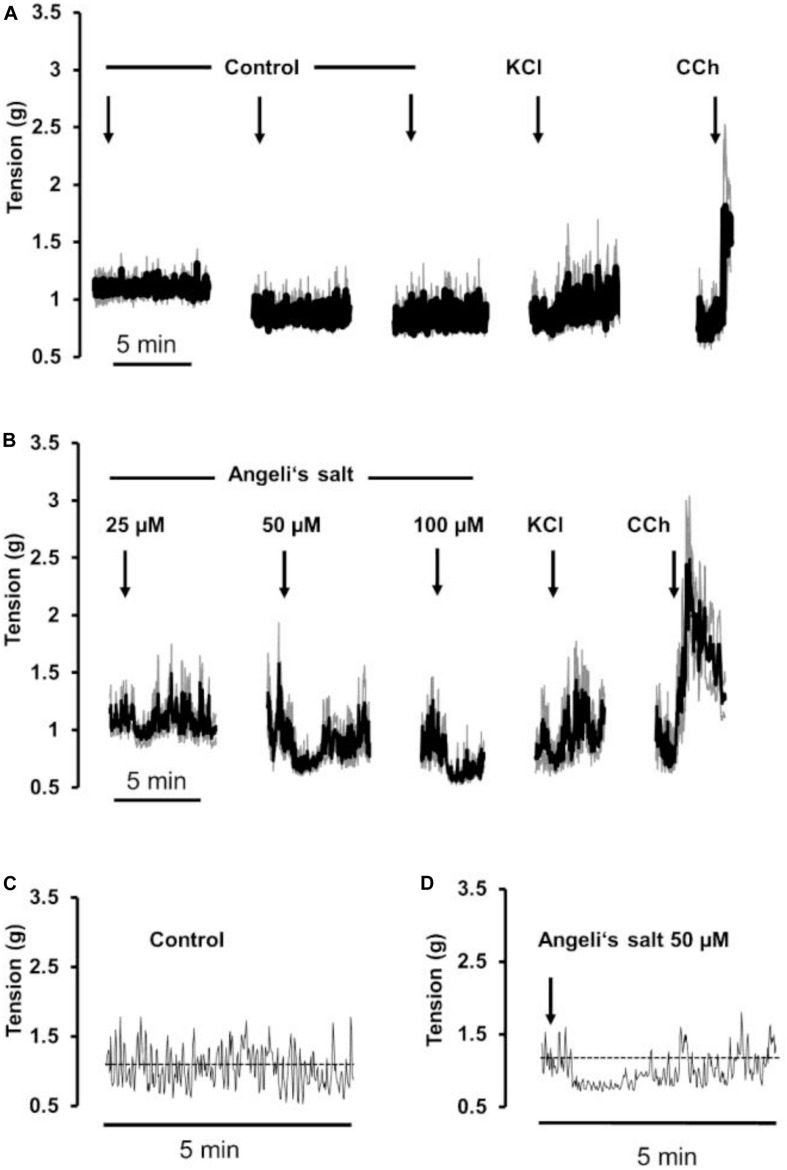
The HNO donor Angeli’s salt (25–100 μM, arrows) induces a relaxation of circular segments of **(B)** rat proximal colon compared to a **(A)** time-dependent control, where only the solvent of Angeli’s salt was administered. Line interruptions are caused by omitting washing periods of about 10 min, where the content of the organ bath was exchanged three times before the next concentration of Angeli’s salt was administered. KCl (30 mM) and carbachol (CCh; 10 μM) were used to check tissue viability. Data in **(A,B)** are means of individual tracings (black lines) ± SEM (gray lines), *n* = 5–6. **(C,D)** are higher magnifications of 5-min intervals of an individual muscle preparation contained in the respective ensemble averages in **(A,B)**. For statistics **(B)**, is plotted in Figures 3E,F.

**FIGURE 3:**
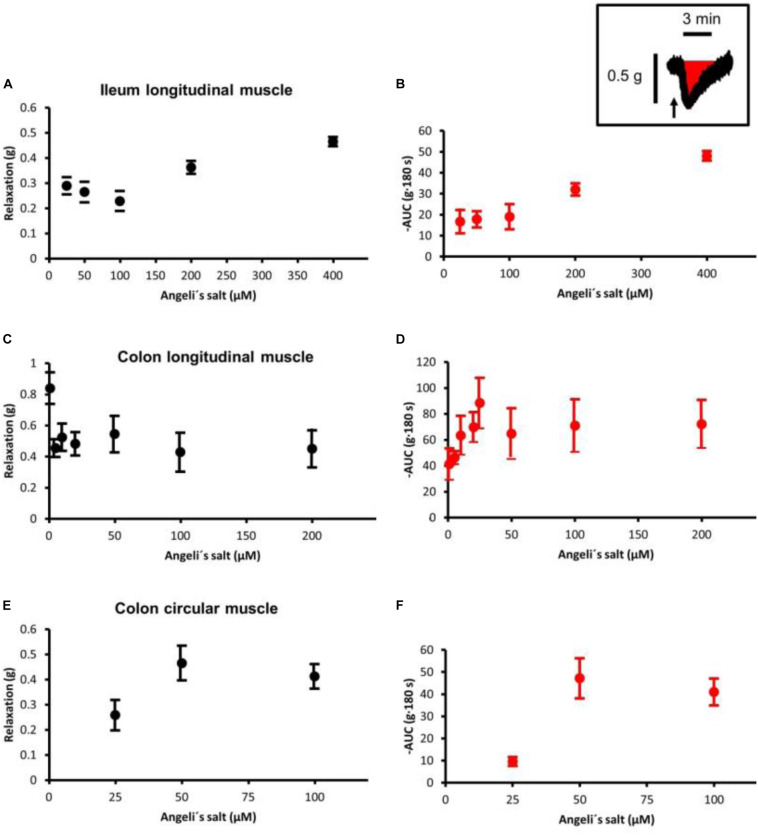
Concentration-dependent relaxation induced by Angeli’s salt in **(A,B)** ileal longitudinal muscle, **(C,D)** colonic longitudinal, and **(E,F)** colonic circular muscle. The relaxing effect is either expressed as maximal reduction in muscle tone (Δ*g*; difference to baseline just prior to administration of the drug **(A,C,E)** or reduction in the area under the curve (AUC) over a 3-min period compared to the 3-min period just prior to administration of Angeli’s salt, as illustrated by the schematic inset where the arrow marks the administration of Angeli’s salt. Concentration–response curves in the colonic longitudinal muscle was constructed from two independent series of experiments (see Figure 1), in which the effect of 1–20 μM and 25–400 μM Angeli’s salt was tested, respectively. Values are means ± SEM, *n* = 6–11.

A relaxing effect induced by Angeli’s salt was also observed in circular muscle preparations from proximal colon (Figure 2B) in comparison to a control series (Figure 2A). The effect of the HNO donor was concentration dependent and showed a maximum at a concentration of 50 μM with respect to the amplitude of relaxation (Figure 3E) and the decrease in the area under the curve over a 3-min period (Figure 3F). Angeli’s salt not only reduced basal muscle tone but also suppressed the phasic contractions as shown when considering records from individual muscle preparations with an extended time scale (Figures 2C,D).

**FIGURE 4:**
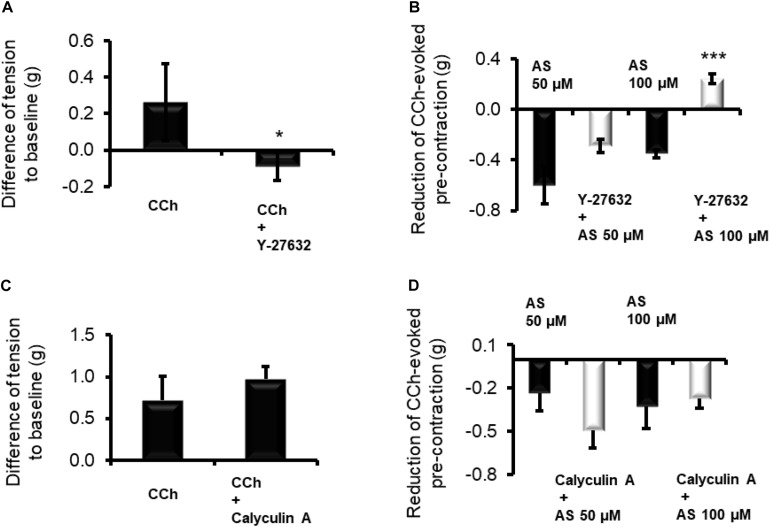
Inhibition of Rho kinases by Y-27632 (10 μM) prevents the contraction induced by CCh (10 μM) of longitudinal muscle strips from **(A)** proximal colon and reduces the relaxation induced by HNO donor Angeli’s salt (AS). Data in **(B)** are expressed as difference between the muscle tone just prior to administration of Angeli’s salt and the muscle tone averaged over a 1-min period starting 1 min after administration of the HNO. Potentiating **(C)** the contractile action of the cholinergic system with calyculin A (100 nM) strengthens or weakens the **(D)** relaxing action of HNO. Values are means ± SEM, *n* = 7–12. *P < 0.05, ***P < 0.001 vs. control in the absence of the corresponding blocker (Mann–Whitney *U*-test). For statistics, see text.

The frequency of phasic spontaneous contractions of longitudinal muscle strips from the colon was significantly (*p* < 0.05, Mann–Whitney *U-*test) reduced by Angeli’s salt. At 50 μM, the HNO donor reduced the frequency from 3.0 ± 0.6 (*n* = 7) cpm to 0.6 ± 0.0 cpm (*n* = 7). Similarly, they were reduced from 1.8 ± 0.6 cpm (*n* = 8) to 0.0 ± 0.0 cpm (*n* = 8) with 100 μM of the donor.

All further experiments were performed with longitudinal muscle strips from the proximal colon and two concentrations of the HNO donor, 50 and 100 μM, were selected for further isometric measurements.

### HNO Interferes With the Cholinergic Pathway for Relaxation

In order to investigate a possible interaction of HNO with MLCP, longitudinal muscle strips of the proximal colon were pre-contracted with 10 μM carbachol, and the amplitude of relaxation caused by HNO was compared in the presence or absence of one of the following smooth muscle contractile apparatus desensitizers calyculin A (inhibitor of the MLCP) or Y-27632 (inhibitor of Rho-kinase). A preliminary test for the validity of Y-27632 (10 μM) – as inhibitor of the cholinergic pathway – was conducted. Indeed, this inhibitor significantly reduced the contraction induced by CCh (Figure 4A). In the presence of Y-27632, the relaxation induced by 50 μM Angeli’s salt was not significantly reduced from −0.59 ± 0.14 g (*n* = 7) to −0.28 ± 0.05 g (*n* = 11), whereas the relaxation induced by 100 μM of HNO donor was reversed into a contraction of +0.24 ± 0.03 g (*n* = 11, measured 1 min after administration of the donor) in comparison to the untreated control, where HNO evoked a relaxation of 0.35 ± 0.03 g (*n* = 7, Figure 4B).

A potentiation of the cholinergic-evoked contraction is expected by inhibiting MLCP, for example with calyculin A. Indeed, the contractile response induced by CCh was slightly increased from 0.71 ± 0.28 g (*n* = 10) to 0.97 ± 0.15 g (*n* = 11) in the presence of 100 nM calyculin A (Figure 4C). MLCP blockade slightly potentiated the relaxing effect of 50 μM Angeli’s salt and attenuated numerically the relaxing response to 100 μM Angeli’s salt; none of these effects, however, reached statistical significance (Figure 4D). However, blocking endogenous ACh effects on the basal tone with 1 μM TTX and 1 μM atropine did not influence Angeli’s salt (50 μM)-induced relaxation as it amounted to −0.49 ± 0.06 g (*n* = 24) and −0.42 ± 0.06 g (*n* = 26) in decrease values or to −120.05 ± 14.80 g⋅180 s (*n* = 24) and −105.05 ± 14.90 g⋅180 s (*n* = 26) for AUC values, in the absence and the presence of both inhibitors, respectively.

### Interplay Between NO, H_2_S, and HNO

**FIGURE 5:**
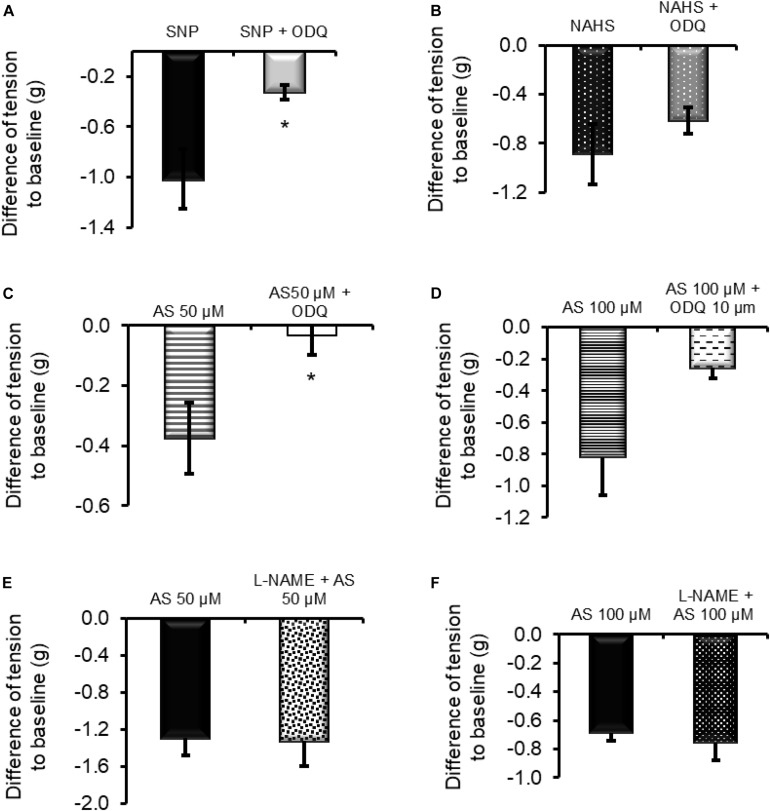
**(A,C)** The relaxing effect of the nitric oxide donor sodium nitroprusside (SNP; 1 mM) and 50 μM Angeli’s salt (AS) was significantly inhibited in the presence of the sGC inhibitor ODQ (10 μM). **(D)** When the concentration of Angeli’s salt was increased to 100 μM, this inhibition failed to reach significance. **(B)** Relaxation induced by the H_2_S donor sodium hydrogen sulfide (NaHS; 100 μM) was unaffected by ODQ. **(E,F)** Inhibition of NOS with L-NAME (200 μM) did not affect HNO-induced relaxation. *n* = 5–9. *P < 0.05, vs. control in the absence ODQ (Mann–Whitney *U*-test). For statistics, see text.

The relaxing properties of NO depend on the activation of sGC. The potential interplay between NO, H_2_S, and HNO may also involve this enzyme. To test this hypothesis, the relaxing action of these three molecules was measured in the presence of the sGC inhibitor ODQ (10 μM). As expected, the inhibitor significantly blocked (Figure 5A) the relaxation induced by the NO donor sodium nitroprusside (SNP, 1 mM) from −1.02 ± 0.23 g (*n* = 6) to −0.32 ± 0.05 g (*n* = 9). Among the other donors, only 50 μM Angeli’s salt was significantly sensitive to the blocker as the relaxation was reduced from −0.37 ± 0.11 g (*n* = 7) to −0.03 ± 0.06 g (Figure 5C, *n* = 8). The relaxation induced by the H_2_S donor sodium hydrogen sulfide (NaHS 100 μM) was not affected by ODQ; it amounted to −0.88 ± 0.24 g (*n* = 5) in the absence and −0.61 ± 0.11 g (Figure 5B) in the presence of the sGC blocker (Figure 5B). Increasing the concentration of AS to 100 μM partially overcomes the ODQ-induced inhibition, suggesting the presence of sGC-dependent and potentially sGC-independent inhibitory pathways (Figure 5D).

To assess the potential importance of endogenously produced NO in HNO-induced relaxation, the relaxing activity of Angeli’s salt was measured after a 30-min pre-incubation period of the tissue with the nitric oxide synthase (NOS) inhibitor L-NAME (200 μM). L-NAME did not affect HNO-induced relaxation (Figures 5E,F) as the maximal relaxation was statistically unchanged from −1.29 ± 0.18 g (control, *n* = 8) to −1.32 ± 0.27 g (test, *n* = 7) for 50 μM Angeli’s salt and from −0.68 ± 0.05 g (control, *n* = 8) to −0.75 ± 0.12 g (test, *n* = 7) for 100 μM Angeli’s salt. Calculating AUC led to the same conclusion. Similar values of AUC were calculated for control and test (in the presence of L-NAME): −163.3 ± 20.2 g⋅180 s (control, *n* = 8) vs. −148.1 ± 19.4 g⋅180 s (test, *n* = 7) for 50 μM Angeli’s salt and −91.6 ± 6.2 g⋅180 s (control, *n* = 8) vs. −77.8 ± 17.3 g⋅180 s (test, *n* = 7) for 100 μM Angeli’s salt.

The impact of endogenously produced H_2_S on HNO action was investigated using inhibitors of the H_2_S-producing enzymes. Thus, amino-oxyacetate (AAOA, 5 mM, cystathionine-β-synthase inhibitor) and β-cyano-L-alanine (CLA, 5 mM, cystathionine-γ-lyase blocker) were used in the presence or absence of the sGC blocker ODQ (10 μM). Inhibition of endogenous synthesis of H_2_S alone led to the reduction in the basal tone by −0.56 ± 0.08 g (AUC, −81.54 ± 13.22 g⋅180 s, *n* = 6) vs. −0.10 ± 0.01 g for control (AUC, −3.85 ± 0.84 g⋅180 s, *n* = 6). This reduction lasted ∼5 min. Preventing H_2_S synthesis, however, only slightly reduced the relaxation induced by 50 μM Angeli’s salt as shown in Table 1. A simultaneous blockade of endogenous synthesis of H_2_S and sGC almost abolished the Angeli’s salt-evoked relaxation (Table 1).

**TABLE 1:** Impact of AOAA/CLA in the presence or not of ODQ on Angeli’s salt-evoked relaxation.

	Δ Force (g)	AUC (g⋅180 s)	*N*
Angeli’s salt alone	−0.70 ± 0.05	−96.4 ± 6.8	6
AOAA/CLA + Angeli’s salt	−0.54 ± 0.07	−71.7 ± 10.0	6
Angeli’s salt alone	−0.97 ± 0.13	−133.0 ± 24.0	5
AOAA/CLA/ODQ + Angeli’s salt	−0.31 ± 0.04^∗∗^	−1.8 ± 1.5^∗∗^	5

*Blockade of endogenous synthesis of H_2_S with both AOAA and CLA (both 5 mM) slightly reduced the relaxation induced by Angeli’s salt (50 μM) in longitudinal muscle from rat colon. Additional blockade of the sGC with ODQ (10 μM) significantly inhibited the response to Angeli’s salt. ^∗∗^P < 0.01 vs. control in the absence of the corresponding blockers (Mann–Whitney U-test) (see Supplementary Figure S2).*

Nitroxyl is highly thiophilic and may interact with thiol groups. In the presence of DTT (500 μM), known to maintain SH groups in reduced state or reduce protein disulfide bonds, the relaxing property of HNO donor was significantly impaired (Table 2). Simultaneous administration of the H_2_S donor (NaHS, 100 μM) and NO donor (SNP, 1 mM) evoked a relaxation that was partially but not significantly sensitive to DTT (Table 2).

**TABLE 2:** Impact of protection of thiol groups on Angeli’s salt- or SNP- and NaHS-evoked relaxation.

	Δ Force (g)	AUC (g⋅180 s)	*N*
Angeli’s salt alone	−1.49 ± 0.25	−186.8 ± 25.9	5
DTT + Angeli’s salt	−0.83 ± 0.07^∗^	−90.4 ± 10.9^∗^	8
NaHS + SNP	−0.73 ± 0.09	−103.3 ± 18.9	5
DTT + NaHS + SNP	−0.59 ± 0.07	−76.4 ± 10.6	8

*The reducing agent DTT (500 μM) significantly inhibited the relaxation induced by Angeli’s salt (50 μM) but only slightly reduced the relaxation induced by NaHS (H_2_S donor; 100 μM) and SNP (NO donor; 1 mM). Longitudinal muscle from rat colon. ^∗^P < 0.05 vs. control in the absence of the corresponding blocker (Mann–Whitney U-test) (see Supplementary Figure S3).*

### Mechanism of Action and Particularity of HNO

#### Calcium Ions Mobilization for Relaxation

H_2_S induces the opening of K_*ATP*_ channels and the closure of voltage-dependent K^+^ channels in guinea pig gastric antrum myocytes (Wang, 2012) or activates small conductance calcium-activated potassium channels (SK_*Ca*_) in mouse colon (Zhao et al., 2009). HNO has been shown to stimulate sarco-endoplasmic reticulum Ca^2+^-ATPase (SERCA) in cardiomyocytes (Irvine et al., 2008). To assess the role of Ca^2+^ in the HNO-induced relaxation, calcium imaging was performed on isolated myocytes of the proximal colon.

**FIGURE 6:**
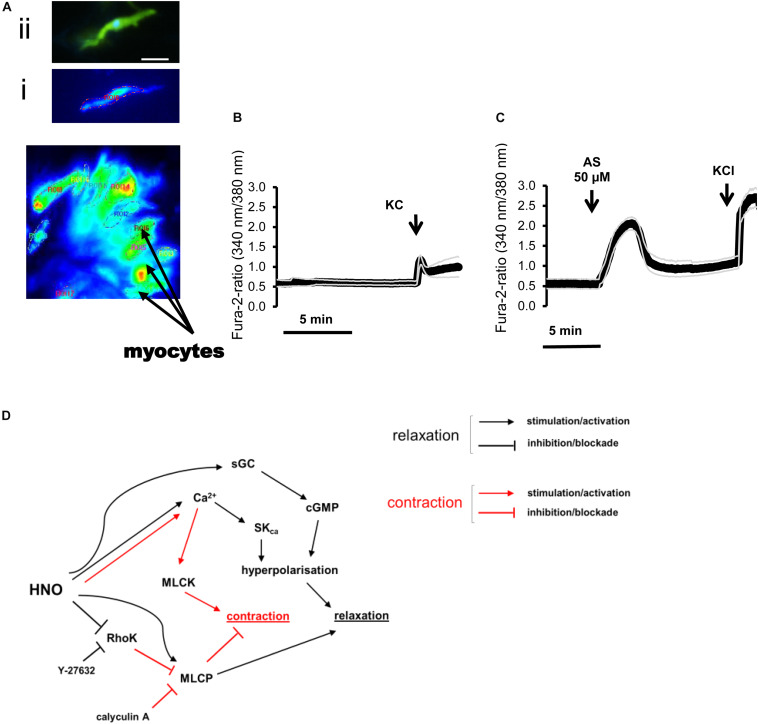
**(A–C)** Changes in the fura-2 ratio evoked by Angeli’s salt (50 μM) in isolated rat colonic myocytes and **(D)** mechanisms underlying relaxation. **(A)** Photograph of isolated myocytes loaded with fura-2. Insets show (i) a fura-2 loaded myocyte that responded to Angeli’s salt and the same cell (ii) after staining of actin filament with phalloidin (green) and nucleus with 4′,6-diamidino-2-phenylindole (DAPI) (blue). Scale bar, 50 μm. **(C)** Angeli’s salt induces an increase in the fura-2 ratio compared to **(B)** a time-dependent control. KCl (30 mM) was used for cell viability control. Values are given as means (symbols) ± SEM (parallel continuous lines), *n* = 6 for the time-dependent control and *n* = 13 for the test group with the HNO donor. For statistics, see text. **(D)** HNO may induce relaxation either directly by activating MLCP or indirectly inhibiting the RhoK. Additional increase in cytosolic Ca^2+^ concentration activates SK_*ca*_ for hyperpolarization and relaxation. Blocking RhoK with Y-27632 shunts HNO effects, inducing a shift toward Ca^2+^-mediated responses, which also activates MLCK for contraction. The main mechanism for HNO-induced relaxation consists in hyperpolarizing myocytes via activation of sGC/cGMP.

In myocytes from the longitudinal layer, Angeli’s salt (50 μM) caused a significant increase in the fura-2 ratio signal from 0.21 ± 0.08 (*n* = 22) to 0.44 ± 0.09 (*n* = 48) (*P* < 0.001, paired *t* test). In myocytes isolated from the circular layer, the response was weaker, as the HNO donor increased the fura-2 ratio from 0.008 ± 0.001 (control; *n* = 13) to 0.057 ± 0.011 (test; *n* = 15) (*P* < 0.001; unpaired *t*-test). A representative result of 6 controls and 13 tests is shown in Figure 6. All the cells tested responded with significant increase in the fura-2 signal to 30 mM KCl, confirming the viability of the cells used (Figures 6B,C).

#### HNO Elicits Membrane Hyperpolarization of Colonic Myocytes

**FIGURE 7:**
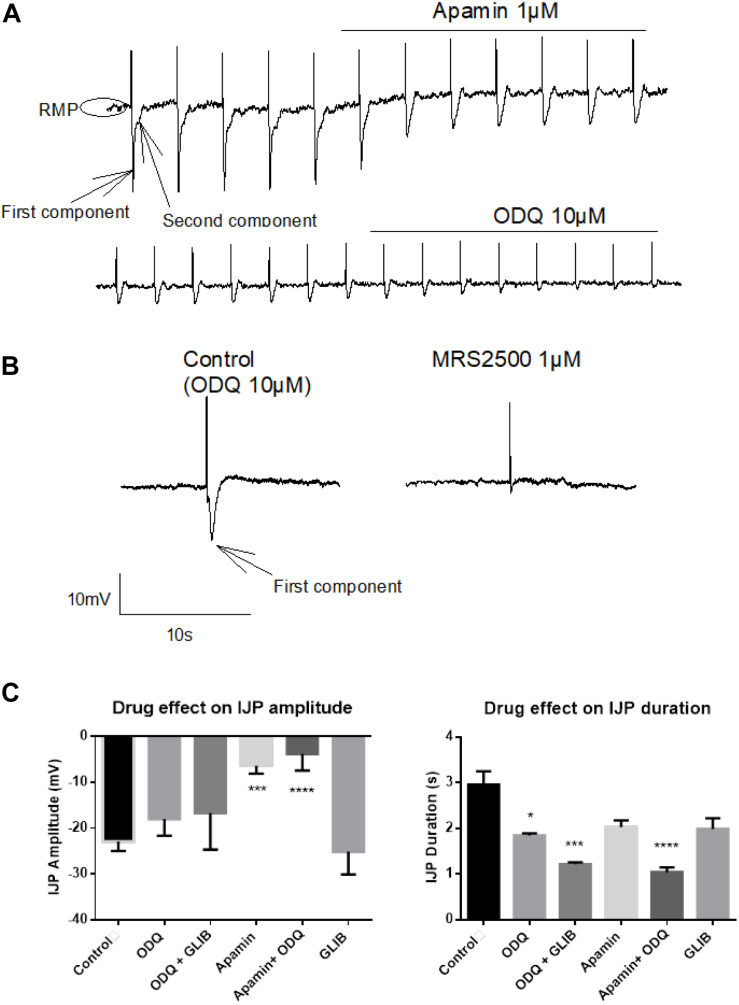
Identification of the fast inhibitory junction potential (IJPf) and sustained IJP (IJPs) evoked by electrical field stimulation (EFS) under non-adrenergic non-cholinergic (NANC) conditions. **(A)** Apamin blocks the IJPf [first component in **(A)**], while ODQ blocks the IJPs [second component in **(A)**]. Apamin induces a shift of the resting membrane potential (RMP) to more positive values. **(A)** The IJPf isolated after suppression of the IJPs by ODQ was also sensitive against MRS2500 **(B)**. **(C)** The amplitude of the IJP was strongly inhibited by apamin; this inhibition was not enhanced, when, in addition, ODQ was administered. In contrast, the duration of the IJP was significantly reduced by ODQ; this effect was significantly enhanced when, in addition, glibenclamide or apamin was present. Concentrations of drugs were apamin 1 μM, glibenclamide 100 μM, and ODQ 10 μM. Values in **(C)** are means ± SEM, *n* = 3–12. *P < 0.05, ***P < 0.001, ****P < 0.0001, vs. control in the absence of any drug (analysis of variance followed by Bonferroni test). For statistics, see text.

**FIGURE 8:**
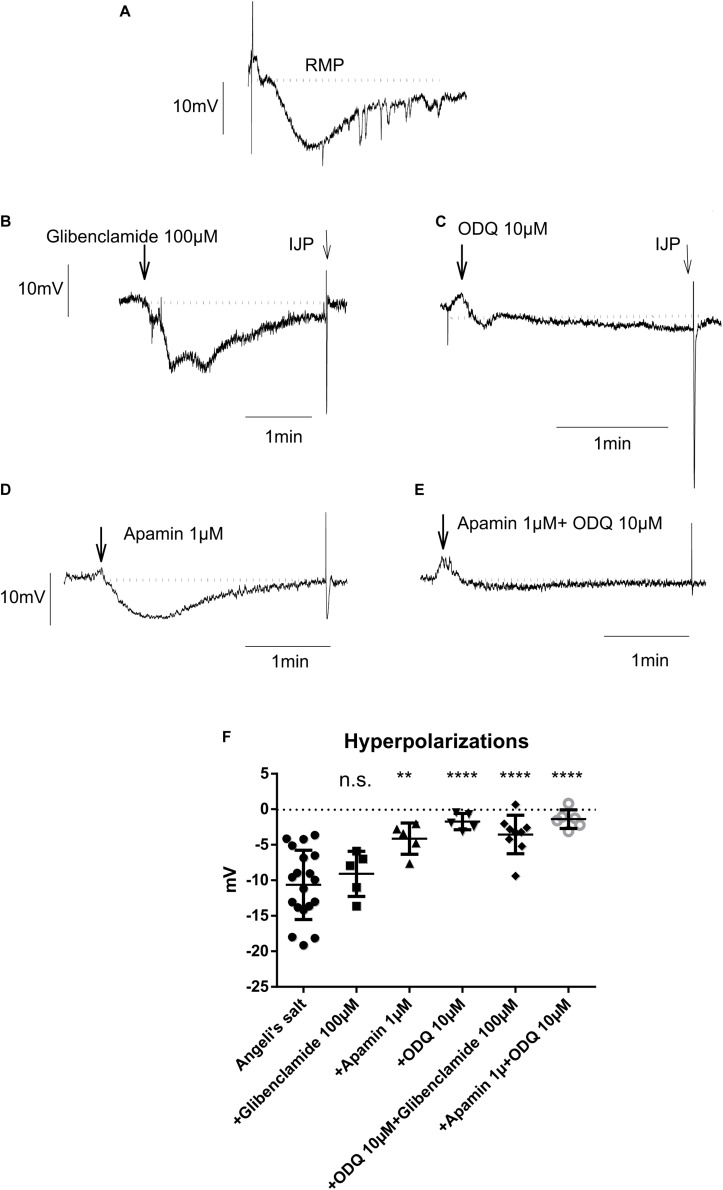
The hyperpolarization caused by the HNO donor Angeli’s salt (25 μM; A, *n* = 19) is insensitive to glibenclamide (**B,F**, *n* = 5), but sensitive to apamin (**D,F**, *n* = 5) or highly sensitive to ODQ alone (**C,F**, *n* = 5) or in combination with apamin (**E,F**, *n* = 6) or glibenclamide (**F**, *n* = 9). The discontinuous horizontal lines represent the resting membrane potential (RMP). Values are means ± SD, *n* values refer to **(F)**, while **(A–E)** are representative original tracings. **P < 0.01, ****P < 0.0001, vs. control in the absence of any drug (analysis of variance followed by Bonferroni test). For statistics, see text.

Accumulation of cytosolic Ca^2+^ as observed in fura-2 experiments (Figure 6) might be responsible for alternative mechanisms of relaxation different from activation of sGC pathways (Figures 1–3). Consequently, HNO inducing relaxation indicates a more complex mechanism. Electrophysiological experiments on myocytes unveiled that the HNO donor Angeli’s salt (25 μM) induced cell membrane hyperpolarization by −10.64 ± 1.11 mV (*n* = 19) from a resting membrane potential of −40 mV (-38 to −45 mV, 95% confidence interval) (Figure 8). The enteric inhibitory neurotransmitters NO, ATP, or related purines and H_2_S, may induce GI myocyte relaxation via activation of sGC/cGMP, or P2Y_1_ purine receptors, or K_*ATP*_, respectively (He and Goyal, 1993; Smith and McCarron, 1998; Gallego et al., 2008; Grasa et al., 2009). To identify the target candidates for HNO-induced relaxation, the hyperpolarization induced by Angeli’s salt was challenged by ODQ (10 μM, inhibitor of the sGC), the bee venom apamin (1 μM, inhibitor of the SK_*ca*_), and glibenclamide (100 μM, inhibitor of K_*ATP*_). As expected, the amplitude of the IJP was strongly reduced by apamin (1 μM) from −22.89 ± 2.08 mV (*n* = 12) to −6.39 ± 0.76 mV (*n* = 5) or any drug combination based on it (apamin + ODQ, from −22.89 ± 2.08 mV, *n* = 12 to −3.83 ± 1.59 mV, *n* = 5) (Figures 7A,C). In the presence of apamin, only the second component associated to the IJPs was recorded (Figure 7A). This second component was sensitive to ODQ (Figure 7A). Accordingly, both MRS2500 and apamin reduced the fast component of the IJP (Figures 7A–C), whereas ODQ (and L-NAME, not shown) reduced the second component of the IJP (Figures 7A,C). Apamin additionally elicits a slight depolarization as the resting membrane potential (RMP) is shifted upwards (Figure 7A). All these results confirm the cotransmission process already described in several species including the rat colon (Grasa et al., 2009) and the human small intestine (Gallego et al., 2014).

#### HNO Targets sGC and SK_*Ca*_

As shown in Figure 8, only glibenclamide did not modify the HNO donor-induced hyperpolarization, as the amplitude of hyperpolarization did not significantly change in the presence of this blocker (-9.08 ± 1.42 mV, *n* = 5) compared to control (Angeli’s salt 25 μM, −10.64 ± 1.11 mV, *n* = 19). Apamin and ODQ significantly reduced the Angeli’s salt-evoked hyperpolarization from −10.64 ± 1.11 mV (*n* = 19) to −4.13 ± 0.98 mV (*n* = 5) for the first inhibitor and from −10.64 ± 1.11 mV (*n* = 19) to −1.73 ± 0.51 mV (*n* = 5) for the second one. In addition, combining ODQ with either glibenclamide or apamin resulted in a very strong inhibition of Angeli’s salt effect (Figure 8F). For the first combination, the hyperpolarization was significantly reduced from −10.64 ± 1.11 mV (*n* = 19) to −3.55 ± 0.90 mV (*n* = 9) and from −10.64 ± 1.11 mV (*n* = 19) to −1.39 ± 0.53 mV (*n* = 6) for the second one. These data lead to the conclusion that the hyperpolarization evoked by HNO is mediated by the activation of SK_*ca*_ channels and sGC, the latter being the predominant pathway.

#### HNO Is a Tone Regulator

Based on the electrophysiological measurements, the question arising as if the relaxation elicited by HNO in organ bath was only relying on the activation of sGC or whether SK_*ca*_ were involved had to be addressed. However, the relaxing action of HNO was not reduced, either in the presence of apamin or MRS2500 (Table 3). This might be due to a major contribution of sGC pathway in inhibitory mechanisms. Accordingly, we tested the effect of apamin in the presence of ODQ. However, as shown in Figure 5C, Angeli’s salt (50 μM) barely relaxes longitudinal muscle strips by −7.36 ± 4.00 g⋅180 s (*n* = 7) and −5.97 ± 2.37 g⋅180 s (*n* = 7), in the absence or presence of apamin; respectively.

**TABLE 3:** Amplitude of Angeli’s salt-evoked relaxation in the presence of apamin and MRS2500.

	Δ Force (g)	AUC (g⋅180 s)	*N*
Angeli’s salt alone	−0.62 ± 0.11	−91.9 ± 19.0	8
Angeli’s salt in the presence of apamin	−0.71 ± 0.12	−100.0 ± 16.8	11
Angeli’s salt alone	−0.62 ± 0.14	−83.4 ± 17.4	5
Angeli’s salt in the presence of MRS 2500	−0.69 ± 0.05	−100.4 ± 8.7	5

*Blockade of SK_*ca*_ with apamin (1 μM) or of purine receptors P2Y1 with MRS2500 (1 μM) does not inhibit the relaxation evoked by Angeli’s salt (50 μM) in longitudinal muscle from rat colon (see Supplementary Figure S4).*

## Discussion

Nitric oxide and hydrogen sulfide are both known to relax intestinal smooth muscle cells (Shuttleworth and Sanders, 1996; Teague et al., 2002). In the present study, we show that also HNO, released by the donor Angeli’s salt, elicits a relaxation of both circular and longitudinal muscle strips from the rat ileum or colon (Figures 1–3) as do NO and H_2_S in different species including rat (Tam and Hillier, 1992; Boeckxstaens et al., 1993; Stark et al., 1993; Teague et al., 2002). This relaxation reaches a maximum within 3 min after application of the donor, which also matches with the profile of NO or H_2_S in their relaxing property (Gallego et al., 2008, 2018). Both the basal tone and the frequency of phasic contractions are reduced by the signaling molecule. The recovery used to occur after 5 min from the onset of the HNO donor (Figure 2D as example). This recovery profile might be due to the short half-life of the donor, which is ∼2–3 min at physiological pH and temperature (Switzer et al., 2009) or the pathways used to achieve the relaxation. In addition, NO and H_2_S present similar periods of action depending on their concentration or the mode of administration or even the donor used.

Neuromuscular transmission regulates smooth muscle contraction by either excitatory transmitters like ACh or inhibitory transmitters like NO. The excitatory neurotransmitter ACh binds to muscarinic G-protein-coupled receptors, initiating a physiological cascade through Gα_*q/*__11_ leading step by step to activation of phospholipase Cβ (PLCβ) and synthesis of IP_3_, which binds to IP_3_R on sarcoplasmatic reticulum for intracellular release of Ca^2+^. In addition to this pathway, receptor-operated (ROC) and stretch-activated (SAC) non-selective cation channels are activated and positively regulate voltage-dependent calcium channels (VDCCs), allowing for Ca^2+^ entry. Another parallel pathway is the G protein regulation of GDP–GTP exchange factor (Rho-GEF), RhoA, and activation of Rho-kinases (RhoK). Activation of RhoK and PKC by diacylglycerol (DAG) or by Ca^2+^ lead to phosphorylation and therefore inactivation of MLCP, the enzyme causing relaxation by dephosphorylating light chain of myosin. Accumulating Ca^2+^ activates the protein calmodulin initiating the activation of kinases such as PKC or myosin light chain kinase (MLCK). The latter phosphorylates light chain of myosin launching cross-bridge cycling (Taylor and Stull, 1988; Sanders, 2008). Relaxation is operated by hyperpolarization counteracting depolarization that resulted from excitation. This can be achieved by activation of Ca^2+^-dependent potassium channels like SK_*ca*_ in smooth muscle cells or alternatively in PDGFRα + cells that might transduce purinergic inputs to smooth muscle cells (Kurahashi et al., 2014). Relaxation can also be achieved by activating MLCP or by extrusion or restoring of cytosolic Ca^2+^.

In the present study, blocking the RhoK by the specific inhibitor Y-27632 (Uehata et al., 1997; Narumiya et al., 2000) blocked the response to CCh (Figure 4A). This confirms that the agonist activates RhoK during muscarinic contraction. In the presence of this inhibitor, the HNO donor-induced relaxation was strongly reduced (Figure 4B), revealing an inactivating property of HNO on the RhoK in the absence of the blocker. The contraction observed with 100 μM (Figure 4B) may indicate a switch in the pathway depending on the concentration of HNO, which, when it is higher, may affect a possible persistent blockade of RhoK or activity of MLCP. A possible reason for this unexpected contraction might be the rise in the cytosolic Ca^2+^ concentration induced by HNO (Figure 6), which might be able to elicit a contraction, when the RhoK is inhibited. A contrasting result was obtained in resistance arteries where NO relaxing activity was potentiated with Y-27632 (Bolz et al., 2003).

On the other hand, inhibition of MLCP activity for example by its specific blocker calyculin A (Ishihara et al., 1989; Bolz et al., 2003) should strengthen the contractile mechanism initiated for example by CCh. A partial effect was observed indeed (Figure 4C). A previous work on arteries also showed increasing contractions upon administration of calyculin A (Ishihara et al., 1989). Thus, pre-incubation with this inhibitor leads to an enhanced pre-contraction status of the tissues. Under this condition, the HNO donor’s relaxing effect was potentiated (Figure 4D) at 50 μM, what is not expected considering a potential inhibitory property of this agent on RhoK as mentioned above. Such a property would have slightly weakened the MLCP inhibition by calyculin A, as MLCP is physiologically inactivated by RhoK, but the potentiation observed would have not occurred. Apparently, a pre-contraction may change the priority of HNO for target selection. This can be observed with a higher concentration of the HNO donor (100 μM, Figure 4D) where the expected reduction in relaxing action of HNO occurs, indicating indeed a direct activation of MLCP or an indirect activation through inhibition of RhoK by HNO. This property activating MLCP is also known for NO (Bolz et al., 2003; Gallego et al., 2016) and H_2_S (Dhaese and Lefebvre, 2009). Figure 6D summarizes the mechanisms of HNO actions on the contractile apparatus. Preventing the effects of endogenous ACh with TTX and atropine could not influence Angeli’s salt-induced relaxation, indicating indeed that HNO may act in non-pre-contracted tissues – that is under normal basal tone – by preferentially selecting sGC/cGMP pathway and secondary activating SK_*ca*_ channels.

It has been shown that NO released by a pure NO donor like diethylamine-NON-Oate (DEA NON-Oate) interacts with H_2_S for HNO production or effects (Eberhardt et al., 2014; Wild et al., 2015; Teicher et al., 2017). This reveals the interplay between both gases NO and H_2_S, contrasting with a potential direct interaction between SNP and H_2_S as shown previously (Filipovic et al., 2013). Combining NO from DEA NON-Oate and H_2_S from Na_2_S synergistically induces release of calcitonin gene-related peptide (CGRP), a substance known to be released during HNO effects (Paolocci et al., 2001; Eberhardt et al., 2014; Wild et al., 2015; Teicher et al., 2017). In trigeminal neurons, Na_2_S/H_2_S is known to be the limiting factor for HNO-like effects (Wild et al., 2015). In the present study, we used a 10:1 concentration ratio for SNP and NaHS in order to release excess NO in the ambient light. Under such conditions, a rapid interaction between SNP and H_2_S should lead to little amounts of HNO. The excessive release of NO from SNP is also expected to interact – as does NO from DEA NON-Oate – with H_2_S. This approach would allow for HNO production/effects either from direct interaction SNP/H_2_S or secondary from NO/H_2_S. Using higher concentrations of H_2_S could help improving HNO production/effects. How exactly the interplay between both gases leads to HNO formation, however, is still subject to debate (Ivanovic-Burmazovic and Filipovic, 2019).

The strong inhibition of the NO-evoked relaxation by ODQ (Figure 5A) was expected since animals that lack GC have no response to NO (Groneberg et al., 2010). Although several interactions between NO and H_2_S have been reported (Szabo, 2017), we did not observe a significant reduction in NaHS response when tissue was incubated with ODQ (Figure 5B). Based on the data obtained with L-NAME, it seems that endogenously synthesized NO does not directly contribute to the relaxation induced by exogenous HNO.

It is known that AAOA may increase spontaneous motility (Gil et al., 2011). However, in the present experiments, we observed a transient reduction in the basal tone when endogenous synthesis of H_2_S was hindered by both AOAA and CLA, indicating a contribution of endogenous H_2_S to the maintenance of basal muscle tone of rat colonic musculature. The relaxation induced by exogenous HNO may be partly potentiated by endogenous H_2_S, as the HNO-evoked maximal relaxation was slightly reduced by the combination AOAA/CLA (Table 1). This indicates a potential switch in the predominant pathway(s) for relaxation depending on the concentration and the nature of the gasotransmitter(s) present *in situ*. Only additional administration of ODQ in the presence of AOAA/CLA abolished AS-evoked relaxation. If a cooperative interplay between NO and H_2_S could lead to endogenous synthesis of HNO, release of HNO could be indirectly sensed by its interaction with thiol groups knowing its high thiophilicity. This thiophilic property was revealed with DTT (Table 2). The relaxation obtained by the simultaneously administration of the NO donor SNP and the H_2_S donor NaHS was partly sensitive to DTT, indicating indeed a partial thiophilic characteristic under both gaseous signaling molecules. Thus, exogenous NO and H_2_S may partially lead to an HNO-like effect. Whether endogenously produced NO cooperates with endogenous H_2_S for HNO-like effects still has to be investigated in more detail, as we used in the present study only one NOS inhibitor (L-NAME) at a single concentration.

In contrast to previous findings showing an activation of the SERCAs in cardiomyocytes (Cheong et al., 2005; Tocchetti et al., 2007; Sivakumaran et al., 2013), cytosolic accumulation of Ca^2+^ upon administration of the HNO donor also makes sense, as this could activate SK_*ca*_ in colonic myocytes, leading to hyperpolarization and therefore to relaxation. The corresponding hyperpolarization was observed under non-cholinergic non-adrenergic conditions to isolate inhibitory responses (Figure 8). Under these pharmacological conditions, the K_*ATP*_ channels known as H_2_S target (Gallego et al., 2008; Zhao et al., 2009) did not play a role in the response to HNO as shown by the missing sensitivity against glibenclamide, the prototypical blocker of this class of ion channels (Figure 8). On the contrary, the HNO-evoked hyperpolarization was dependent both on sGC and SK_*ca*_ as the hyperpolarization was strongly reduced by the corresponding inhibitors ODQ and apamin, respectively (Figure 8). One possibility is that HNO might be acting on enteric inhibitory neurons causing the release of purines acting on P2Y1 receptors that activate SK_*Ca*_ channels. However, MRS2500 did not modify the mechanical relaxation induced by HNO (Table 3), and therefore, it is possible that the effect of HNO is not on enteric inhibitory neurons but a direct effect on post-junctional cells. These results are consistent with calcium measurements in colonic myocytes. Whether these mechanisms are located only in smooth muscle cells or other post-junctional cells such as ICC or PDGFRα + cells is currently unknown. As PDGFRα + cells may express more SK_*ca*_ than smooth muscle cells (Koh et al., 1997; Kurahashi et al., 2011; Baker et al., 2015), the involvement of such cells when considering the muscle strips and not isolated myocytes cannot be excluded.

All these results indicate a clear activation of SK_*ca*_ and sGC/cGMP pathway by HNO in colonic myocytes. Acute analgesic effects of the HNO donor Angeli’s salt are prevented by treatment with ODQ, glibenclamide, or KT5823 (inhibitor of protein kinase G), suggesting a mechanism of action via activation of the cGMP/PKG/K_*ATP*_ pathway (Longhi-Balbinot et al., 2016). A further pathway that can be activated by HNO after interaction with sGC is the release of CGRP and activation of K_*ATP*_ channels as shown during vasodilatation (Favaloro and Kemp-Harper, 2007). Activation of sGC by HNO, as it is the case with NO, may occur via coordination to the ferrous enzyme as shown on purified bovine lung sGC (Miller et al., 2009). It is also known that the oxidized ferric enzyme is not influenced by HNO (Miller et al., 2009). Due to its high thiophilicity, HNO modifies regulatory thiols on sGC, leading to inhibition of the enzyme activity (Miller et al., 2009). Low concentrations of Angeli’s salt up to 10 μM activate sGC from bovine lung, whereas high concentrations from 100 μM began inhibiting enzyme activity (Miller et al., 2009). It has been shown that ODQ inhibits sGC by oxidation of the ferrous to the ferric heme (Zhao et al., 2000). In Figure 5C, ODQ significantly reduced Angeli’s salt effect, revealing the capacity of HNO to activate sGC more likely at the ferrous heme. At 100 μM of the HNO donor (Figure 5D), ODQ failed to inhibit the relaxation. Here, we could speculate that Angeli’s salt still can activate the heme counteracting ODQ action. HNO induces intradisulfide formation in PKGIα, therefore activation of the kinase leading to vasorelaxation in mesenteric arteries *in vitro* and arteriolar dilatation *in vivo* in mice. This activation of the kinase is similar to binding of cGMP (Donzelli et al., 2017). NO activates the sGC/cGMP pathway, but other types of K^+^ channels, namely, the KNO1, KNO2, and the BK channels (Koh et al., 1995).

We can conclude that HNO presents similarities with NO and H_2_S when it comes to mechanisms of action or pathways regulated. All three gaseous signaling molecules cause GI tract relaxation. HNO causes relaxation via activation of the sGC/cGMP pathway leading to hyperpolarization (like NO), activation of SK_*Ca*_ channels (like H_2_S), activation of MLCP (like both NO and H_2_S), and inhibition of RhoK. It also corrects impaired pathways and induces a fine tuning between motility-regulating properties of NO and H_2_S. HNO may also present a motility “sensor” property. It interferes with disulfide bonds, and the amplitude of its response may be dependent on the balance –S–S–/SH at its targets. In our previous study focusing on colonic epithelial ion transport (Pouokam et al., 2013), interaction of HNO with thiols was also observed, as the prosecretory property of HNO was sensitive to L-cysteine. This property was Ca^2+^ dependent and mediated by activation of the secretory machinery consisting among others of the basolateral Na^+^-K^+^-ATPase, the Ca^2+^-dependent K^+^ and the K_*ATP*_ channels. The present work shows an involvement of Ca^2+^ in HNO-induced relaxation. In contrast to the dependence of the prosecretory property on eicosanoids revealed by the sensitivity of the HNO-induced secretion to 1 μM indomethacin (Pouokam et al., 2013), in the present study, the same concentration of indomethacin does not reduce HNO-induced relaxation. Indeed, similar values of force or AUC were recorded in the absence and presence of 1 μM indomethacin: 0.74 ± 012 g (*n* = 11) or −101.20 ± 18.89 g⋅180 s (*n* = 11) for control and 0.72 ± 0.13 g (*n* = 12) or −103.15 ± 20.45 g⋅180 s (*n* = 12) for test in the presence of the inhibitor. These observations indicate a predominant role of eicosanoids in the secretory process but their secondary or minor role in mediating HNO effects on the motility.

HNO alone may act as a hybrid signaling molecule between NO and H_2_S with more regulatory properties and some particularities. Consequently, HNO appears to be an excellent candidate for substituting NO and or H_2_S in the therapy of diverse GI tract disorders. Nitroxyl-prodrugs (temposil, dipsan) have been used so far as alcohol deterrent agents or undergo clinical trials (CXL 1427) (Cowart et al., 2015). Investigating on the appropriate *in situ* concentration and the ideal therapeutic window as it is known for each drug (Paolocci et al., 2007) could be a great benefit for the GI tract.

HNO relaxes the gastrointestinal tract musculature by hyperpolarization of myocytes via activation of the sGC/cGMP pathway similarly to NO, inhibiting the RhoK and activating MLCP as do both NO and H_2_S. HNO also increases cytosolic Ca^2+^ for activation of SK_*Ca*_, contributing to hyperpolarization. Using two different rat strains led to same conclusions. The effects of HNO imply contribution of thiols and do not depend primarily on eicosanoids. To our knowledge, this study is the first one showing the effects of HNO on the intestinal motility.

Although the pharmacological tools and the techniques used isometric force measurements, electrophysiology, Ca^2+^ imaging, and immunostaining help understanding the mechanistic of HNO actions, there are some limitations that we will need to tackle in further studies. Indeed, there is a need to better understand the mechanism of action of HNO on the contractile apparatus, with focus on the single components of this machinery. Sarcoplasmatic/endoplasmatic reticulum calcium ATPase (SERCA) 2 gene encodes two spliced variants SERCA2a and SERCA2b. The SERCA2a is specific for heart tissue (Khan et al., 1990; Spencer et al., 1991; Lytton et al., 1992) but can be expressed in low amounts in smooth muscle (Vandecaetsbeek et al., 2009), whereas SERCA2b is mostly expressed by smooth muscle cells and non-muscle cells (Spencer et al., 1991; Lytton et al., 1992) SERCA2b apparently presents a twofold higher affinity for cytosolic Ca^2+^ ions and a lower maximal turnover rate (Vandecaetsbeek et al., 2009). Other studies, however, point out a contradictory property, a rather lower turnover rate for Ca^2+^ transport and ATP hydrolysis (Lytton et al., 1992). The lower expression of SERCAa in smooth muscle cells may account for potential difference in Ca^2+^ signaling compared to cardiomyocytes. However, we cannot confirm this since we did not look for the expression of SERCA2 in colonic myocytes used in our study for calcium imaging. We used isolated myocytes deprived from vascular tissue. In addition, how HNO activates sGC or modulates cGMP still has to be investigated. Endogenous production of HNO remains a striking issue, as very small amounts if produced are difficult to detect and so a potential interplay of endogenous NO and H_2_S remains questionable. Developing more HNO donors with longer half-life is needed to explore the multitude of HNO effects.

## Data Availability Statement

The datasets generated for this study are available on request to the corresponding author.

## Ethics Statement

The animal study was reviewed and approved by Animal welfare officer of the Justus Liebig University (administrative number 577_M) and the Ethics Committee of the Universitat Autònoma de Barcelona.

## Author Contributions

EP designed the experiments. MG-S, MJ, and EP performed the *in vitro* experiments, analyzed the recordings, and wrote the manuscript.

## Conflict of Interest

The authors declare that the research was conducted in the absence of any commercial or financial relationships that could be construed as a potential conflict of interest.

## Abbreviations

AOAA: amino-oxyacetate
CLA: β -cyano-L-alanine
DTT: 1,4-dithiothreitol
GI: gastrointestinal
L-NAME: N_ω_-nitro-L-arginine methylester hydrochloride
MLCP: myosin light chain phosphatase
ODQ: 1H-[1,2,4]oxadiazolo [4,3-a] quinoxalin-1-one
RhoK: Rhokinase
RMP: resting membrane potential
sGC: soluble guanylate cyclase.
