# Supplementary figures and images for: Mechanisms Associated to Nitroxyl (HNO)-Induced Relaxation in the Intestinal Smooth Muscle
Source: Front Physiol. 2020 Jun 3;11:438. doi: 10.3389/fphys.2020.00438 (PMC7283591; doi:10.3389/fphys.2020.00438)

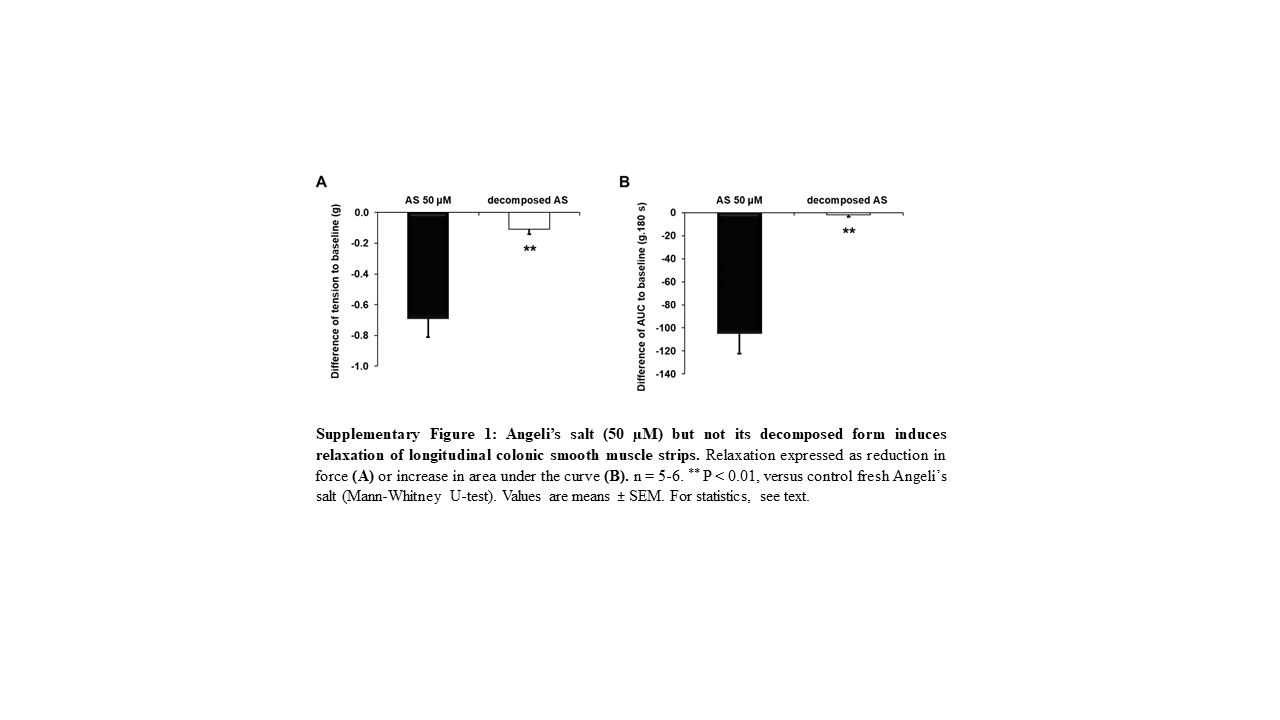

Supplement: Supplementary file 1 [file Image_1.tif]

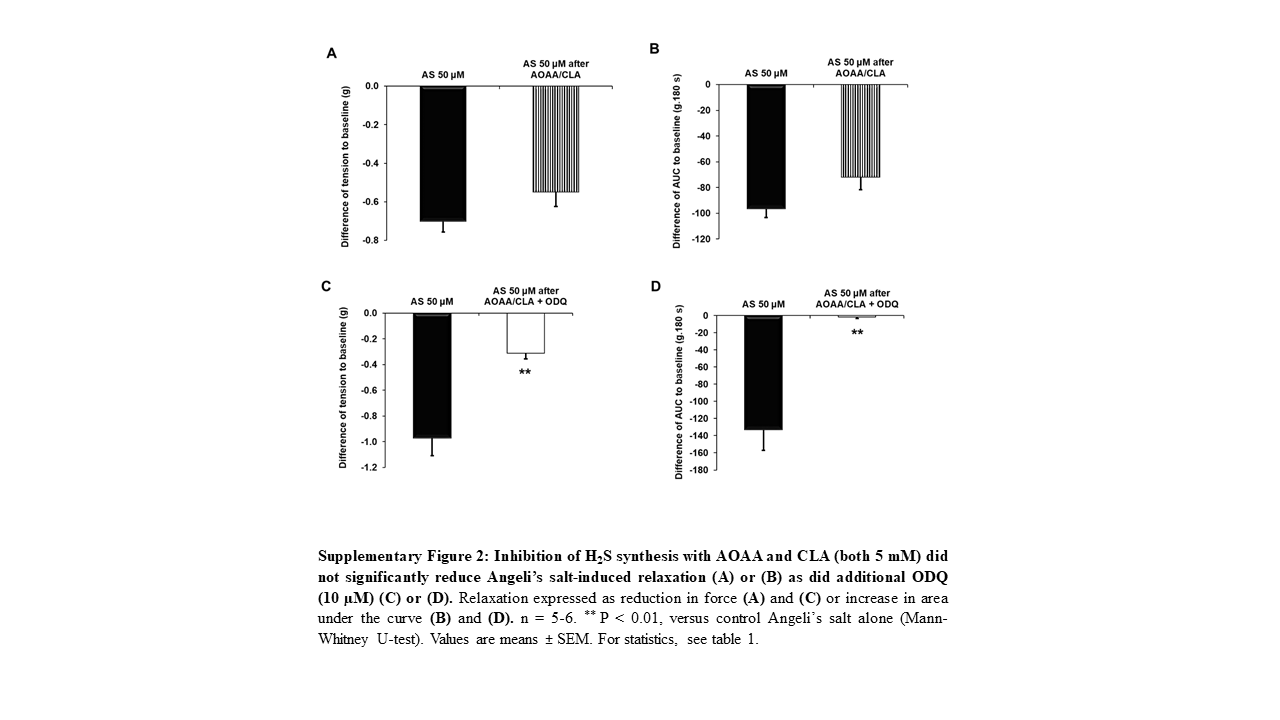

Supplement: Supplementary file 2 [file Image_2.tif]

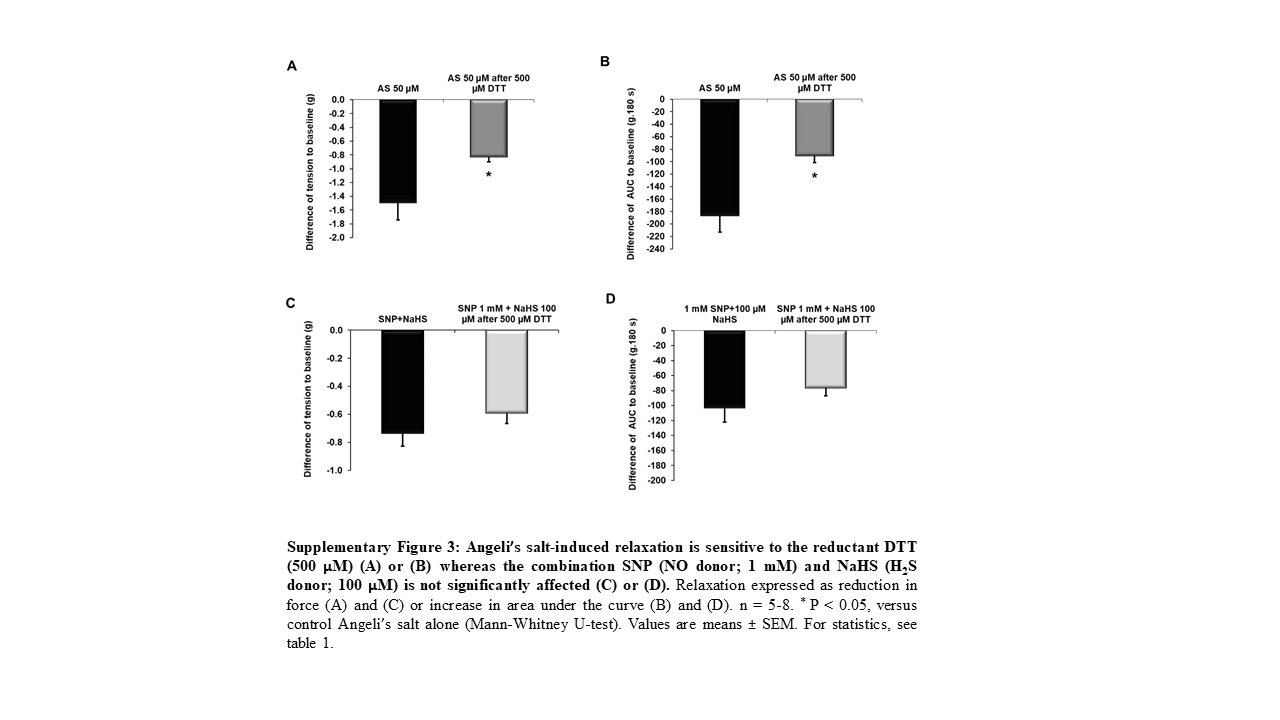

Supplement: Supplementary file 3 [file Image_3.tif]

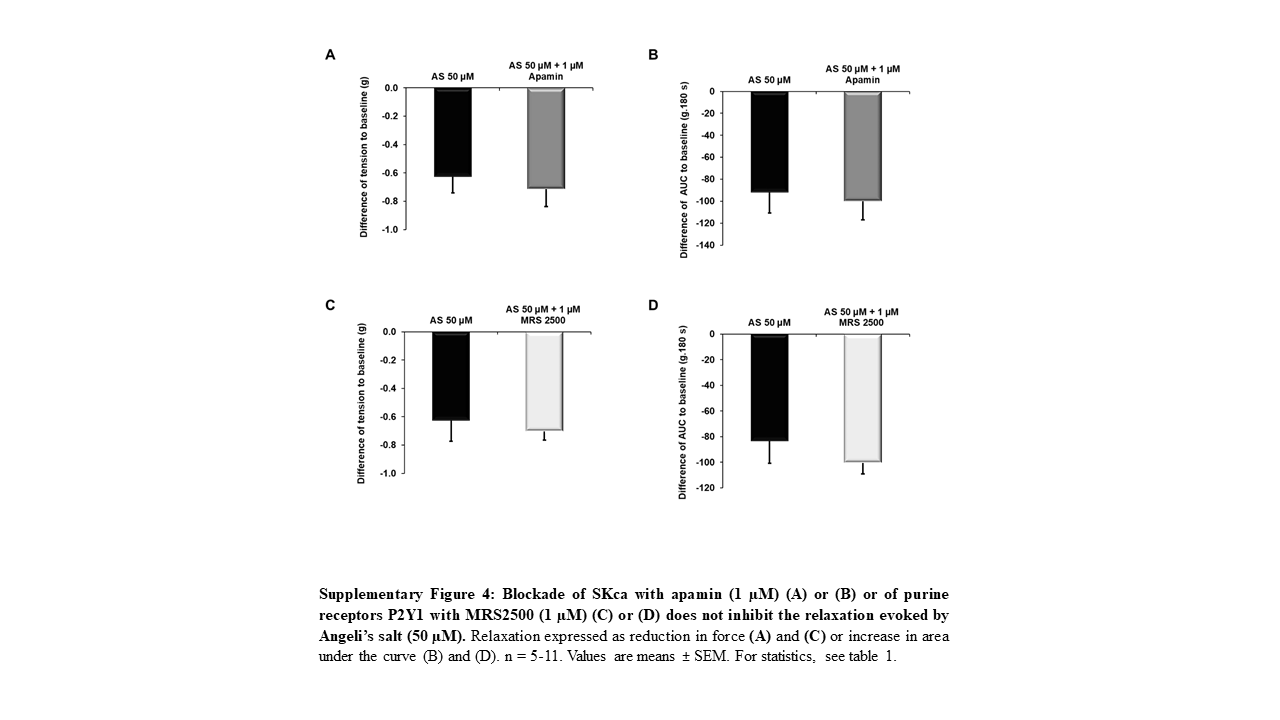

Supplement: Supplementary file 4 [file Image_4.tif]
